# Inhibition of HER2-integrin signaling by Cucurbitacin B leads to *in vitro* and *in vivo* breast tumor growth suppression

**DOI:** 10.18632/oncotarget.1743

**Published:** 2014-02-25

**Authors:** Parul Gupta, Sanjay K. Srivastava

**Affiliations:** ^1^ Department of Biomedical Sciences and Cancer Biology Center, Texas Tech University Health Sciences Center, Amarillo, TX, USA; ^2^ Cancer Preventive Material Development Research Center, College of Korean Medicine, Department of Pathology, Kyunghee University, 1 Hoegi-dong, Dongdaemun-ku, Seoul, South Korea

**Keywords:** Breast cancer, in vivo, ITGB4, ITGA6, Cucurbitacin B

## Abstract

HER2, an oncogenic receptor is overexpressed in about 25-30% of breast cancer patients. HER2 has been shown to play role in tumor promotion by having cross-talk with multiple oncogenic pathways in cancer cells. Our results show that Cucurbitacin B (CuB), a triterpenoid steroidal compound inhibited the growth of various breast cancer cells with an IC50 ranging from 18-50nM after 48 and 72 h of treatment. Our study also revealed the significant inhibitory effects of CuB on HER2 and integrin signaling in breast cancer. Notably, CuB inhibited ITGA6 and ITGB4 (integrin α6 & integrin β4), which are overexpressed in breast cancer. Furthermore, CuB also induced the expression of major ITGB1and ITGB3, which are known to cause integrin-mediated cell death. In addition, we observed that TGFβ treatment resulted in the increased association of HER2 with ITGA6 and this association was inhibited by CuB treatment. Efficacy of CuB was tested *in vivo* using two different orthotopic models of breast cancer. MDA-MB-231 and 4T-1 cells were injected orthotopically in the mammary fat pad of female athymic nude mice or BALB/c mice respectively. Our results showed that CuB administration inhibited MDA-MB-231 orthotopic tumors by 55%, and 4T-1 tumors by 40%. The 4T-1 cells represent stage IV breast cancer and form very aggressive tumors. CuB mediated breast tumor growth suppression was associated with the inhibition of HER2/integrin signaling. Our results suggest novel targets of CuB in breast cancer *in vitro* and *in vivo*.

## INTRODUCTION

Breast cancer is the most commonly diagnosed malignancy in women. Studies suggest that around 232,340 new cases will be diagnosed and around 39,620 women will die due to breast cancer by the end of 2013 [1]. The HER2/neu is a proto-oncogene, which is amplified in several neoplasms like breast, salivary gland, stomach, kidney and lung [2-7]. It is overexpressed in about 30% of breast cancer patients, which leads to poor clinical outcomes [2-7]. HER2 is a mitogenic tyrosine kinase receptor, known to induce transforming signals and hence directly promotes cell proliferation [8]. The HER2 expression can lead to impaired DNA repair [8], angiogenesis [9] and metastasis [10]. Furthermore, HER2 has been shown to interact with several other growth promoting mechanisms like TGFβ, estrogen receptor, FOXO1A, Src kinases, ILK and integrins to reinforce oncogenesis and metastasis [11-16]. Trastuzumab (herceptin) is a specific antibody for HER2 that is used against HER2+ breast cancer. But studies indicate limitations of trastuzumab therapy due to cardio-toxicity and resistance in patients [17].

Integrins is a large family of cell adhesion receptors, which also perform other diverse functions like cell survival and motility. Various integrins have been implicated in tumor cell survival, proliferation and metastasis [18, 19]. Importantly, two major integrin heterodimers ITGAVB3 (αVβ3) and ITGA6B4 (α6β4) have been found to play specific role in breast cancer progression and metastasis [20-26]. There is a strong evidence that cross-talk between HER2 and ITGA6B4 promotes tumor aggressiveness [16, 27-29]. ITGB4 (Integrin β4) contributes to HER2 up-regulation in mammary tumors suggesting a strong correlation between HER2 and integrins in mammary tumor development and progression [30]. Identification of novel agents that can target both HER2 and integrin signaling could be beneficial for the treatment of breast cancer overexpressing HER2.

Cucurbitacins are triterpenoid steroidal compounds mainly present in the Cucurbitaceae family of plants [31]. Several evidences suggest the cytotoxic properties of Cucurbitacin B (CuB) through cell cycle arrest and STAT3 inhibition in pancreatic cancer and hepatocellular carcinoma [32-35]. However, the exact mechanism of CuB is not clear.

The objective of the current study was to evaluate whether CuB could suppress breast cancer growth by inhibiting HER2/integrin signaling. Our results showed that CuB inhibits HER2 expression in breast cancer cells through ILK1 and YB-1 in both *in vitro* and *in vivo* models. In addition, it was observed that CuB inhibits ITGA6B4 (integrin α6β4) signaling and the subsequent cross-talk with HER2. Our study provides a novel insight into the mechanism of action of CuB along with evidence for the role of HER2-integrin signaling in breast cancer.

## RESULTS

### CuB inhibits breast cancer cell growth by inducing apoptosis

**Figure 1:**
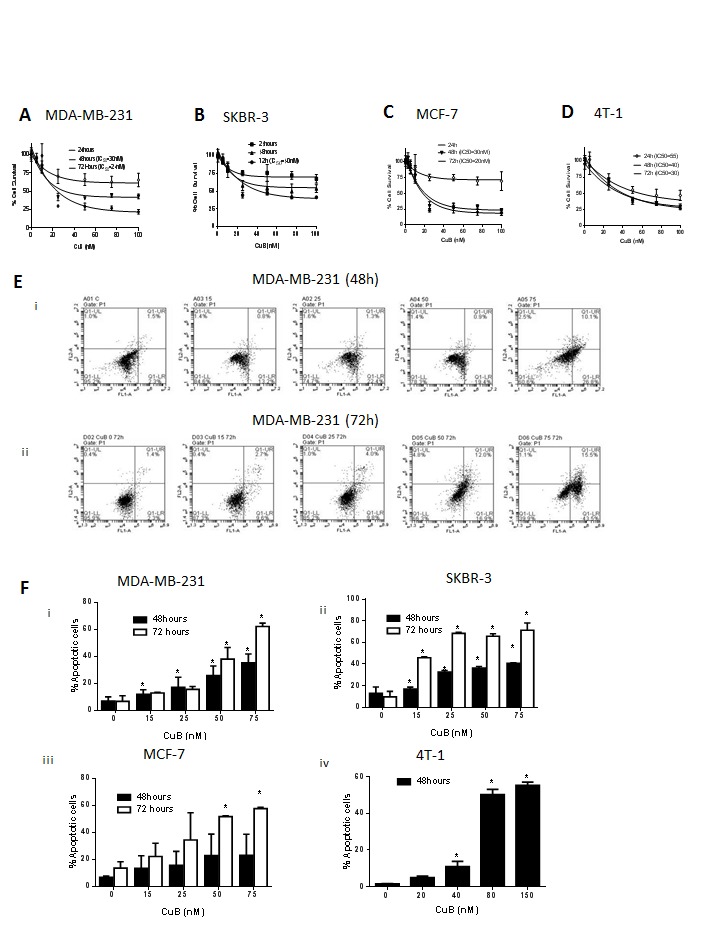
CuB induces cell death in breast cancer cells (A) MDA-MB-231, (B) SKBR3 (C) MCF-7 & (D) 4T-1 cells were treated with increasing concentrations of CuB for 48h or 72. Cell survival was measured with sulforhodamine B assay to estimate the IC_50_ values. The experiments were repeated at least three times with 8 replicates in each experiment. Apoptosis induction was measured in cells treated with various concentrations of CuB using Annexin V-FITC assay and Accuri C6 flowcytometer. (E) Representative figure of apoptosis in MDA-MB-231 cells treated with CuB (i) 48h and (ii) 72h. (F) Concentration dependent apoptosis induction in various cell lines after 48 and 72h of CuB treatment (i) MDA-MB-231 (ii) SKBR3 (iii) MCF-7 and (iv) 4T-1 cells. Each experiment was repeated more than three times independently. *Statistically significant when compared to control (p<0.05)

Considering breast tumor heterogeneity, we used four different cell lines with diverse phenotype and genotype. Treatment of MDA-MB-231, SKBR3, MCF-7 and 4T-1 breast cancer cells with increasing concentrations of CuB significantly reduced the survival of these cells in a concentration and time-dependent manner with an IC50 ranging between 18 – 50nM after 48 and 72h treatment (Fig. 1A - D). Previous studies reported significantly high IC50 of CuB in normal mammary epithelial cell lines as compared to SKBR3 breast cancer cells [36]. To confirm the non-toxic effects of CuB, we evaluated its toxicity in a normal human melanocyte epithelial (PIG1) cells. Our results showed that the viability of PIG1 cells treated with CuB was least affected as compared to the viability of cancer cells (Suppl. Fig 1A). For example, treatment of with 50nM CuB for 72h inhibited the growth of PIG1 cells by 20-30% only. However, the growth of cancer cell lines like SKBR3, MDA-MB-231, MCF-7 and 4-T1 were inhibited by 50-70% after treatment with CuB under similar conditions (Suppl. Fig 1B). These results along with previous observations suggest that CuB is relatively non-toxic to the normal cells at the concentrations required for inhibiting the growth of cancer cells.

**Figure 2:**
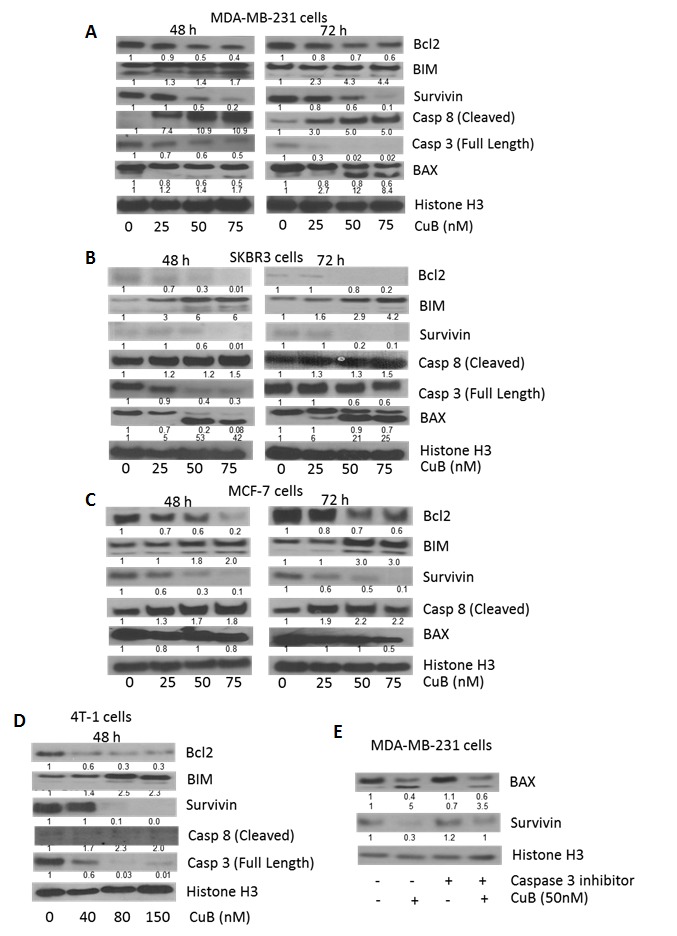
Induction of caspase mediated apoptosis by CuB: (A) MDA-MB-231 and (B) SKBR3 (C) MCF-7 and (D) 4T-1 cells were treated with varying concentrations of CuB for 48 or 72h Representative blots showing the concentration dependent effect of CuB on Bcl2, BIM, Survivin, Caspase 8 (cleaved) and Caspase 3 (full length). (E) MDA-MB-231 cells were treated with Q-VD-OPH, a specific inhibitor for caspase 3, 1h prior to CuB treatment. The cells were collected after 48h treatment and cell lysates were analyzed for BAX cleavage using western blot technique. Histone H3 was used as a loading control. Each experiment was repeated at least three times independently.

To explore the mechanism of the growth inhibitory effects of CuB, MDA-MB-231, SKBR3 and MCF-7 cells were treated with 0, 15, 25, 50 and 75nM CuB for 48 or 72h. 4T-1 cells required higher concentration of CuB for induction of apoptosis and the molecular changes hence were treated with 0, 20, 40, 80 and 150nM CuB for 48h. The cells were analyzed for apoptosis using Annexin V assay. As shown in Fig. 1E & F, 75nM CuB treatment for 72h induced apoptosis in about 80% of SKBR3 cells and about 60% in MDA-MB-231, MCF-7 and 4T-1 cells. To further investigate the mechanism of apoptosis in CuB treated breast cancer cells, western blot analysis was performed. The western blot data of whole cell lysates from CuB treated MDA-MB-231, SKBR3, MCF-7 and 4T-1 cells showed significant down-regulation of Bcl2 and survivin (Fig. 2A-D). Although, SKBR3 cells expressed low constitutive levels of Bcl2 and survivin, the extent of apoptosis induced by CuB was comparable with other cell lines indicating the role of multiple pathways in CuB mediated cell death. On the other hand, expression of pro-apoptotic BIM was up-regulated along with cleavage of Caspase 8 (Fig. 2A-D). We were unable to detect the cleaved fragments of Caspase 3 and hence looked at full length Caspase 3 (pro-caspase 3). The expression of full-length Caspase 3 decreased in response to CuB treatment in all the cell lines tested indicating apoptosis (Fig. 2A-D). These observations indicate the concentration-dependent induction of apoptosis by CuB in breast cancer cells.

Interestingly, we observed cleavage of BAX by CuB treatment. Expression of BAX generally increases in response to apoptotic stimuli leading to caspase activation [37]. BAX cleavage mediated by caspases or calpains is known to play a much stronger role in apoptosis [38, 39]. We observed that CuB treatment caused a concentration dependent cleavage of BAX at both 48h and 72h time points in MDA-MB-231 and SKBR3, while no cleavage was observed in MCF-7 cells (Fig. 2A-C). To prove it further, MDA-MB-231 cells were treated with caspase 3 inhibitor along with CuB treatment. The results from this experiment showed that caspase 3 inhibitor significantly blocked CuB mediated cleavage of BAX (Fig. 2E). Since MCF-7 is a caspase 3 null cell line, no cleavage of BAX was observed. These results indicate a direct role of caspase 3 in BAX cleavage in our model [40].

### CuB treatment suppresses HER2 signaling

**Figure 3:**
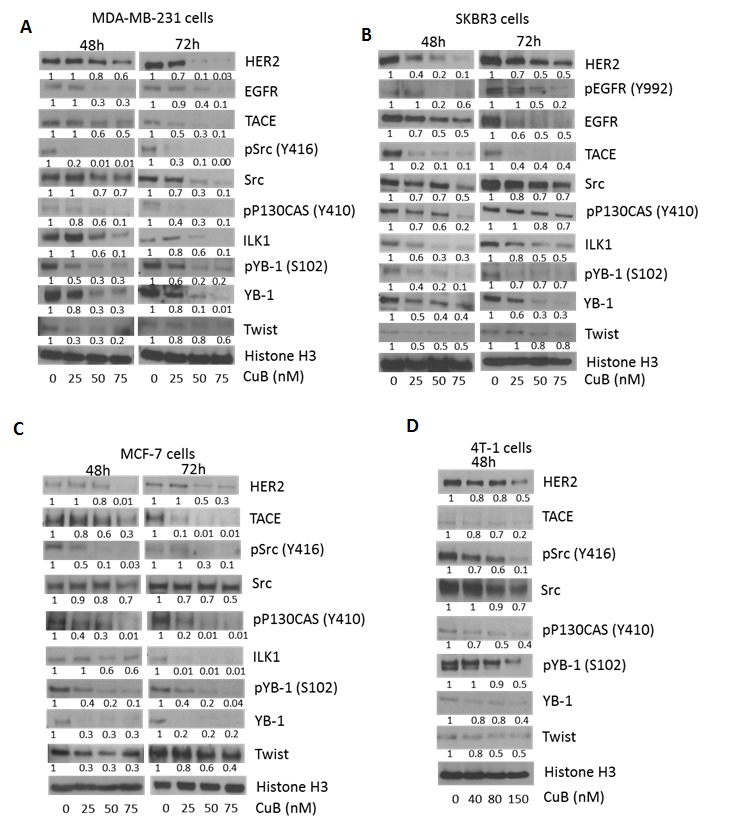
Suppression of HER2 and associated signaling by CuB treatment: (A) MDA-MB-231 (B) SKBR3 (C) MCF-7 and (D) 4T-1 cells were treated with varying concentrations of CuB for 48 or 72h Representative blots showing concentration dependent effect of CuB on HER2, EGFR, phospho EGFR (Y992) TACE, phosho-Src (Y416), Src, phospho-P130CAS (Y410), ILK1, phosphor-YB1 (S102), YB-1 and Twist. Histone H3 was used as a loading control. Each experiment was repeated atleast three times independently.

To determine the mechanism of cell death induced by CuB treatment, cell lysates were analyzed by western blotting. Our results showed that treatment of MDA-MB-231, SKBR3, MCF-7 and 4T-1 cells with CuB after 48 or 72h of treatment significantly reduced HER2 and EGFR expression in a concentration-dependent manner (Fig. 3A-D). Although both HER2 and EGFR were reduced after 72h of CuB treatment, effect of CuB was more pronounced at 48h in SKBR3 and MCF-7 cells. CuB inhibited HER2 in all the cell lines irrespective of ER or p53 status. Phosphorylated EGFR was undetectable in MDA-MB-231, MCF-7 and 4T-1 cells. However, CuB treatment significantly suppressed the phosphorylation of EGFR at Y-992 after 72h treatment in SKBR3 cells (Fig. 3B). CuB treatment also substantially reduced the expression and phosphorylation of Src at Y-416 along with decreased phosphorylation of p130CAS at Y-410. The inhibitory effects of CuB on the phosphorylation and expression of Src varied with duration of treatment in MDA-MB-231, SKBR3 and MCF-7 cells (Fig. 3A-D). However, longer treatment did not cause any further suppression of phosphorylated p130CAS in any cell line. p130CAS is a downstream effector molecule of Src. Src plays a vital role in the cross-talk of HER2 with integrins in breast tumors [16]. Furthermore, we observed suppression of TNFα-converting enzyme protease (TACE) by CuB treatment (Fig. 3A-D). The 72h treatment with CuB caused more TACE inhibition as compared to 48h treatment in MDA-MB-231 and MCF-7 cells (Fig. 3A & C). TACE has been implicated in the activation of EGFR and HER2 by TGFβ [16, 41]. Since CuB down-regulated HER2 expression, it was important to test the effect of CuB on the regulatory molecules of HER2. As shown in Fig. 3A-D, treatment of cells with CuB substantially reduced the expression of ILK1 and Twist. It was observed that in MCF-7 cells, CuB treatment reduced ILK1 expression more at 72h treatment as compared to 48h. TWIST suppression was more pronounced at 48h treatment with CuB in all the three cell lines. Furthermore, suppression of YB-1 expression as well its phosphorylation at S-102 was also observed with CuB treatment (Fig. 3A-D). The ILK1/YB-1/Twist signaling axis has been known to regulate HER2 expression in breast cancer cells [15]. Our results showed that higher HER2 inhibition was observed at 48h in only those cell lines where TWIST along with ILK or YB1 were also inhibited. These observations suggest that inhibition of HER2 by CuB was associated with the inhibition of ILK1/YB-1 signaling.

### CuB suppresses ITGA6B4

**Figure 4:**
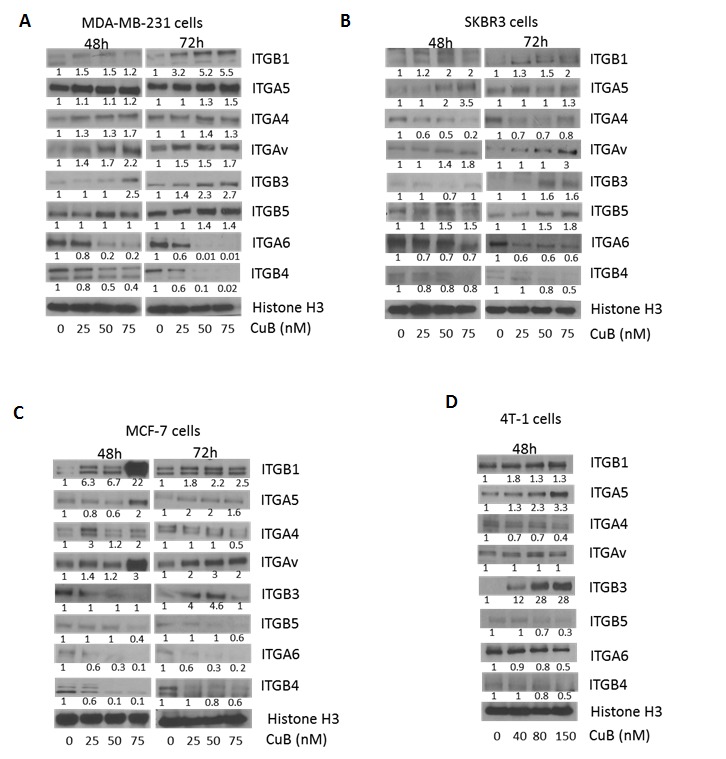
CuB suppresses integrin α6/β4 expression: (A) MDA-MB-231 (B) SKBR3 (C) MCF-7 and (D) 4T-1 cells were treated with different concentrations of CuB for 48 or 72h The proteins in cell lysates were separated using western blot. The blots were immune-probed for the integrins ITGB1 (integrin β1), ITGA5 (integrin α5), ITGAV (integrin αv), ITGB3 (integrin β3), ITGB5 (integrin β5), ITGA6 (integrin α6), ITGB4 (integrin β4) and Histone H3 was used as the loading control. Each experiment was repeated atleast three times independently.

To evaluate the role of CuB on integrin signaling, MDA-MB-231, SKBR3, MCF-7 and 4T-1 cells were treated with CuB for 48 and 72h. Our results showed that CuB significantly suppressed the expression of ITGA6 and ITGB4 as compared to other integrins in the cell lines tested in a time-dependent manner (Fig. 4A-D). The other integrins were either not affected at all or very modest changes were observed by CuB treatment. These results indicate that CuB causes specific inhibition of ITGA6 and ITGB4, which interestingly are overexpressed in breast cancer and interacts with HER2. On the other hand, the pro-apoptotic ITGB1 and ITGB3 were induced in a time-dependent manner in MDA-MB-231 and SKBR3 cells after treatment with similar concentrations of CuB. Although, significant induction of ITGB1 was observed at 48h treatment with CuB in MCF-7 cells, cleavage of caspase 8 or apoptosis did not correlated as compared with other cells. The induction of pro-apoptotic ITGB1 and ITGB3 by CuB treatment suggests activation of integrins mediated cell death by CuB in MDA-MB-231, SKBR3 and 4T-1 breast cancer cells.

### CuB inhibits TGFβ mediated interaction of HER2 with ITGA6

**Figure 5:**
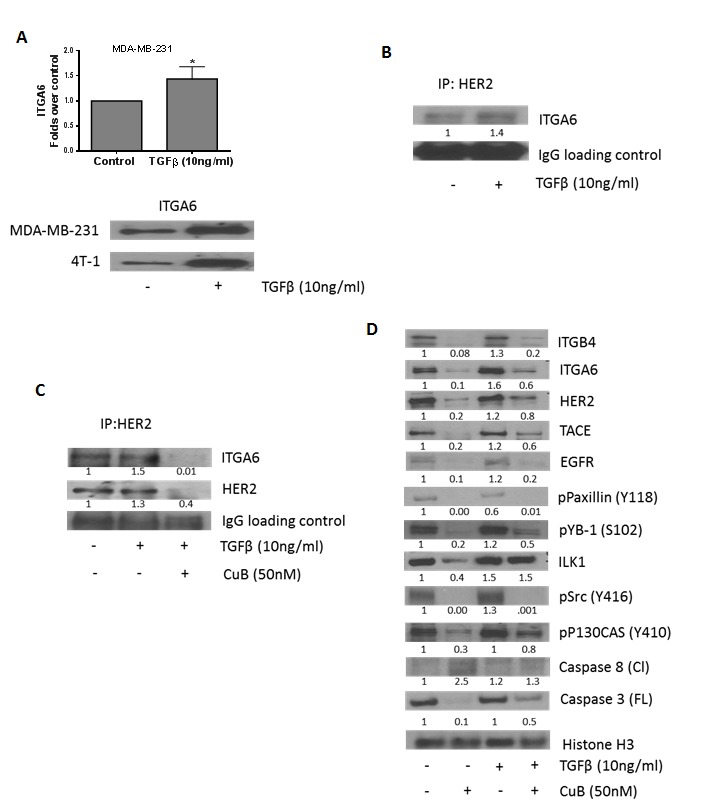
CuB inhibits TGFβ-mediated interaction of HER2 and integrin α6 (A) TGFβ treatment induces expression of integrin α6 in MDA-MB-231 and 4T-1 cells. (B) HER2 was immune-precipitated from MDA-MB-231 cell lysate with or without TGFβ treatment after 48h and the protein was separated using western blot. The blots were probed for ITGA6. IgG was used as the loading control. (C) HER2 was immune-precipitated from cell lysates of MDA-MB-231 cells with or without treatment with TGFβ and CuB for 48h. The protein was separated using western blotting and the blots were probed for ITGA6 and HER2. IgG was used as the loading control (D) MDA-MB-231 cells were treated alone or in combination with TGFβ and CuB for 48h. The proteins in cell lysates were separated using western blot. The blots were probed for ITGB4, ITGA6, HER2, TACE, EGFR, phospho paxillin (Y118), phospho YB1 (S102), ILK1, phospho Src (Y416), phospho P130CAS (Y410), Caspase 8 (cleaved) and Caspase 3 (full-length). Histone H3 was used as a loading control. Each experiment was repeated atleast three times independently.

Since TGFβ is known to activate HER2/integrin signaling, we evaluated the interaction of HER2 with integrins. TGFβ treatment significantly increased the expression of ITGA6 by about 1.5 fold in MDA-MB-231 and 4T-1 cells (Fig. 5A). Interestingly, when HER2 was immune-precipitated from TGFβ treated cells, we observed that TGFβ treatment increased the expression of ITGA6 associated with HER2 (Fig. 5B). However, this interaction was inhibited by CuB treatment (Fig. 5C). These observations propose a novel mechanism of tumor promotion by TGFβ by increasing the association between ITGA6 and HER2, possibly due to induction of ITGA6. Our results thus provide a novel insight into TGFβ-mediated physical interaction between HER2 and ITGA6. However, further studies are required to confirm the detailed mechanism and implications of this interaction.

To determine the effect of CuB on integrin signaling in presence of TGFβ, MDA-MB-231 cells were treated with CuB (50nM) after treatment with TGFβ (10ng/ml). Our results indicate that TGFβ significantly blocked CuB-mediated inhibition of ITGA6, HER2, TACE, ILK1 and phosphoP130CAS (Y410) (Fig. 5D).

### Inhibition of integrin signaling by CuB

**Figure 6:**
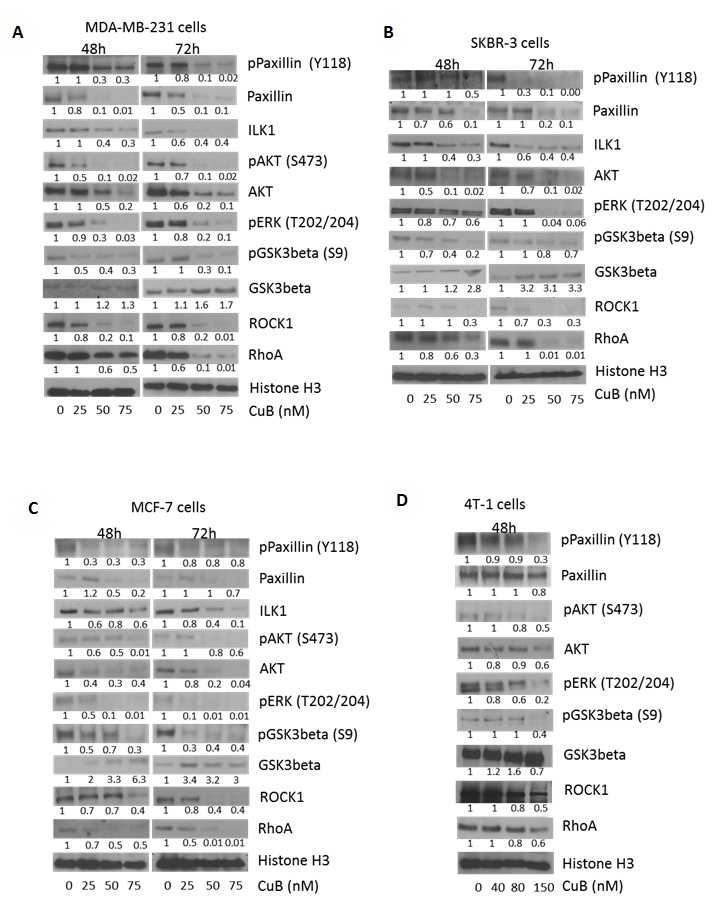
CuB inhibits integrin signaling cascade: (A) MDA-MB-231 (B) SKBR3 (C) MCF-7 and (D) 4T-1 cells were treated with various concentrations of CuB for 48 or 72h The cell proteins were separated using western blot. The blots were immune-probed for phospho paxillin (Y118), paxillin, ILK1, phospho Akt (S473), Akt, phospho ERK (T202/204), phospho GSK3β (S9), GSK3 β, ROCK1 and RhoA. Histone H3 was used as the loading control. Each experiment was repeated at least three times independently.

Next we tested the effects of CuB on downstream signaling of integrins in breast cancer cells. MDA-MB-231, SKBR3, MCF-7 and 4T-1 cells were treated with various concentrations of CuB for 48 or 72h. As shown in Fig. 6A-D, CuB treatment suppressed the expression and phosphorylation of paxillin at Y-118 (Fig. 6A-D). A significant down-regulation of ILK1 was also observed by CuB treatment. ILK1-paxillin complex regulated by integrins serves as a major mechanism for various cell survival processes. CuB also inhibited the phosphorylation of AKT at S-473 and ERK at T-202/204 (Fig. 6A-D). Both AKT and ERK are important downstream targets of ILK1-paxillin complex. Consistently, CuB treatment suppressed the phosphorylation of GSK3β at S-9 while enhancing its protein level in a concentration and time dependent manner (Fig. 6A-D). AKT is known to negatively regulate GSK3β in cancer cells. CuB also suppressed ROCK1 and RhoA kinases which play important role in cell motility (Fig. 6A-D). Both the kinases are known to be activated by integrins and are important targets of Akt.

### CuB inhibits breast tumor growth in mice

**Figure 7:**
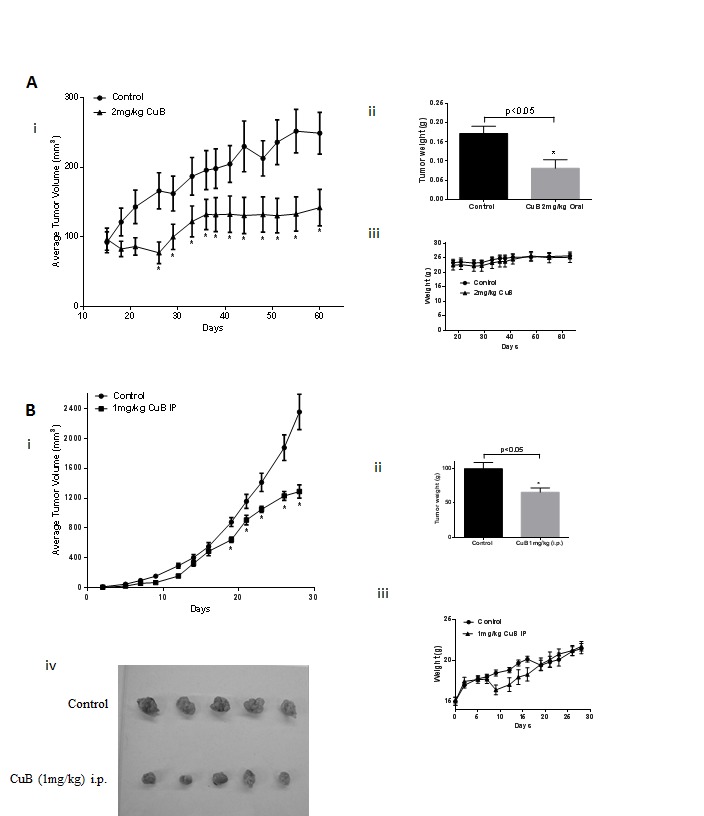
CuB suppresses breast tumor growth (A) About 5X10^6^ MDA-MB-231 cells were orthotopically implanted in the mammary fat pad of female athymic nude mice. Once each mouse had a tumor of about 50-80mm^3^, 2mg/kg CuB was administered by oral gavage every day. Tumors were measured periodically and each mouse was weighed every week. Effect of CuB on (i) tumor volume, (ii) tumor weight and (iii) mice weight. (B) About 0.1X10^6^ 4T-1 cells were orthotopically implanted in the mammary fat pad of female BALB/c mice. Once each mouse had a tumor of about 30-50mm^3^, 1mg/kg CuB was injected by i.p route every third day. Tumors were measured periodically and each mouse was weighed every week. Effect of CuB on (i) tumor volume, (ii) tumor weight (iii) mice weights and (iv) representative tumor size after dissection.

To test the efficacy of CuB *in vivo*, two breast tumor experiments were performed. In the first experiment, 5X10^6^ MDA-MB-231 cells were injected orthotopically in the mammary fat pads of female athymic nude mice. Once each mouse attained palpable tumors, mice were randomly divided into two groups with ten mice in each group. Treatment group received 2mg/kg CuB everyday by oral gavage. The tumor growth was monitored by measuring tumor volumes periodically. Our results showed that oral gavage of CuB significantly reduced the growth of breast tumors (Fig. 7A). At day 60 of the treatment, tumor volume in the treated group was reduced by 55% as compared to the control group [249.5±30.3mm^3^ vs 111.3±18.4mm^3^; (n=10)] (Fig. 7Ai). The weight of the MDA-MB-231 tumors dissected from treated mice was about 53% less than the weight of tumors from control mice (Fig. 7Aii). The weight of mice did not change significantly with oral gavage, indicating no apparent systemic toxicity in CuB-treated mice (Fig. 7Aiii). In the other experiment, 0.1X10^6^ 4T-1 breast cancer cells were injected in the mammary fat pads of female BALB/c mice. 4T-1 cells are highly aggressive and considered to be late stage breast cancer. Once each mouse attained palpable tumors, mice were segregated into two groups with eight mice in each group. 1mg/kg CuB was given intraperitoneally, every third day to the mice of treatment group and the growth of the tumors monitored. The growth of tumors in CuB treated group was reduced significantly as compared to control group (Fig. 7B). For example, at day 28 of the treatment, 4T-1 tumor volume in the treated group was reduced by 39% as compared to control group [2355.3±236.4mm^3^ vs 1446.7±142.6mm^3^; (n=8)] (Fig. 7Bi). Similarly, the weight of the tumors in treated mice was reduced by 27% (Fig. 7Bii & iv). A transitory reduction in weight was observed in the mice receiving i.p. administration of CuB as compared to control, which ameliorated with time (Fig. 7Biii). Since the weight of the mice dropped initially after intraperitoneal administration, we decided to evaluate the mice of treated group for any signs of gross toxicity. The organs of control and treated mice were weighed once the experiment was terminated. No change in the weight of organs like liver, spleen, kidney, lungs, brain and heart were observed in CuB treated mice as compared to control mice (Table 1). The enzymatic activities of AST, ALT and LDH were also evaluated in the plasma of control and CuB treated mice. Although, no significant change in the activity of ALT and AST was observed, interestingly, plasma LDH level was reduced by 24.3% in the mice of CuB treated group (Table 2). The exact reasons behind the decrease in LDH level by CuB treatment was not clear and require further studies.

**Table 1:** Body weight and organ weight data from control and CuB treated mice

Treatment	body weight (g)	Liver (g)	Liver/BW^a^	Spleen (g)	Spleen/Bw^a^	Kidney (g)	Kidney/Bwa	Lungs (g)	Lungs/Bw^a^	Brain (g)	Brain/Bw^a^	Heart (g)	Heart/Bw^a^
Control	20.79±0.86	1.60±0.11	0.076±0.00	0.94±0.11	0.04±0.00	0.41±0.02	0.02±0.00	0.30±0.02	0.01±0.00	0.41±0.01	0.02±0.00	0.15±0.01	0.01±0.00
CuB 1mg/kg i.p.	19.15±1.17	1.53±0.10	0.08±0.00	0.7±0.10	0.04±0.00	0.43±0.02	0.02±0.01	0.33±0.02	0.02±0.00	0.38±0.03	0.02±0.01	0.17±0.02	0.01±0.01

a Ratio of specific organ to body weight (BW)

**Table 2:** The enzymatic activities of AST, ALT and LDH in the plasma of control and CuB treated mice

	AST	ALT	LDH
Control	108.59±8.12	13.51±1.6	260.91±29.1
CuB 1mg/kg i.p.	90.54±19.5	18.41±1.6	197.25±47.35

**Figure 8:**
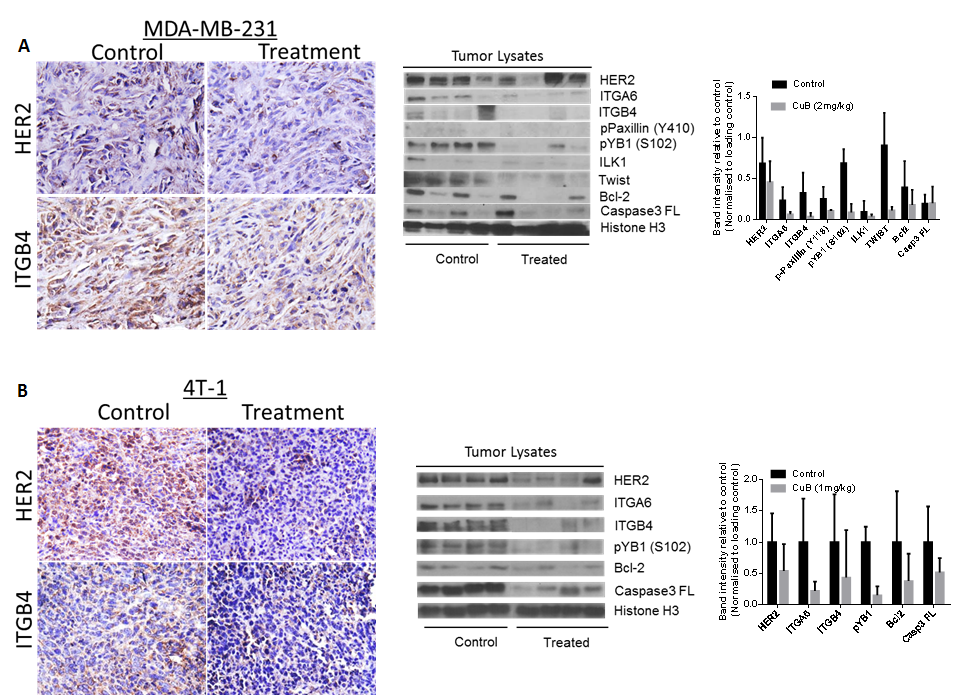
*In vivo* suppression of HER2-Integrin signaling by CuB treatment: Tumors were removed after the termination of the experiment Tumors were minced, lysed and analyzed for HER2, ITGA6, ITGB4, phospho paxillin (Y118), phospho YB1 (S102), ILK, Twist, Bcl2 and Caspase 3. Blots were stripped and re-probed with Histone H3 antibody to verify equal protein loading. (A) Each lane of blot represents a tumor sample from individual mouse. The blots were quantitated, normalized with loading control and represented as bars. (B) Representative images from the control and CuB treated mice i) MDA-MB-231 tumors stained for HER2 and ITGB4 ii) 4T-1 tumors stained for HER2 and ITGB4

To determine if the tumor growth suppression *in vivo* by CuB was associated with the inhibition of HER2/integrins, immunohistochemistry (IHC) of tumor sections and western blot of the tumor tissue lysates was performed. The tumor sections from CuB treated mice showed reduced expression of HER2 and ITGB4 in MDA-MB-231 as well as 4T-1 tumor sections (Fig. 8 A & B). Our western blot results also confirmed that the expression of HER2, ITGA6 and ITGB4 were suppressed in the tumors of CuB treated mice (Fig. 8A & B). Tumor lysates from CuB treated mice also revealed suppression of phospho YB-1 (S-102) and Twist, which are known to regulate HER2 expression in breast cancer cells. In addition to suppression of HER2-associated signaling, CuB treatment also inhibited integrins' downstream signaling molecules like phospho paxillin (Y118) and ILK1 in the tumors. The induction of apoptosis by CuB treatment was confirmed through down-regulation of Bcl-2 and pro-caspase 3 in the tumors of CuB treated mice. Taken together, these results indicate that the breast tumor growth suppression by CuB *in vivo* was associated with the inhibition of HER2/integrin signaling validating our *in vitro* observations.

**Figure 9:**
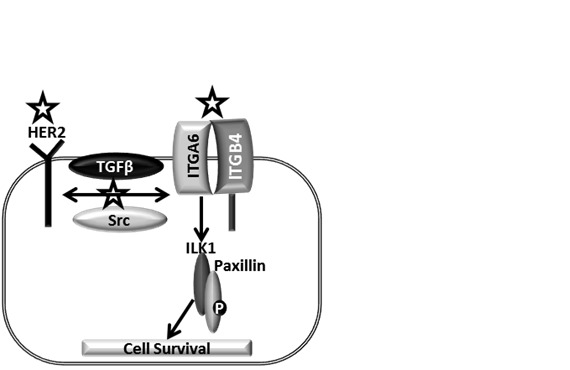
Model mechanism of CuB in breast cancer cells: HER2, an important oncogene enhances breast cancer progression HER2-integrin cross-talk mediated by TGFβ or Src further promotes breast cancer. The complex of ILK1 and Paxillin orchestrates the downstream effects of integrins. Symbol represents the targets inhibited by CuB.

## DISCUSSION

HER2 overexpression is observed in about one-fourth of breast cancer patients and is a major cause for poor prognosis [3]. The crosstalk of HER2 with several other growth signaling pathways results in extremely aggressive form of cancer, imparting high resistance to conventional therapeutic modalities [42]. Our study identified a unique HER2-integrin signaling axis in breast cancer that was inhibited by CuB, a novel steroidal triterpenoid compound [31].

Several previous studies used CuB extracted from plants instead of using commercially available pure compound to evaluate cytotoxicity. Tannin-Spitz et. al. used the plant extract to evaluate the cytotoxic properties of CuB [43]. This study showed an IC50 of 8μM in MDA-MB-231 cells after 48h of treatment. On the other hand, Promkan et al. observed an IC50 of 70μM (38.9μg/ml) after 48h of treatment [44]. In contrast to these studies, our study showed significantly reduced effective concentrations of CuB. The IC50 of CuB after 72h of treatment ranged between 18nM and 50nM in most of the breast cancer cell lines tested. Our results are in agreement with the IC50 observed by Wakimoto et. al., who used pure CuB [45]. Hence, differences in the IC50 of CuB in the published literature could be attributed to the purity of CuB and cancer model. We observed that CuB was effective in 4T-1 cells at slightly higher concentrations as compared to other cell lines. This could be due to several reasons: 4T-1 cells overexpress p-glycoprotein (P-gp) and hence is resistant to several chemotherapeutic agents [46]. It is possible that CuB could be the substrate for P-gp, leading to a requirement of higher concentrations of CuB in this cell line. Secondly, 4T-1 is a very aggressive and resistant cell line as compared to other cells used in our study, thus needing more CuB to suppress its growth.

Our studies showed that CuB treatment induced caspase mediated apoptosis in breast cancer cells. Apoptosis was confirmed through cleavage of caspase 8 and inhibition of full-length caspase 3. It is known that Bcl2 and survivin play a crucial role in caspase inhibition and their inhibition leads to activation of caspases. We observed significant reduction of these proteins by CuB treatment. Although, SKBR3 cells expressed low constitutive levels of Bcl2 and survivin, the apoptosis inducing effect of CuB was not compromised in SKBR3 cells, suggesting the role of other intermediary molecules. BAX is an important protein that regulates the activation of caspases and accumulation of BAX leads to apoptosis [37]. On the other hand, several studies have shown that cleavage of BAX mediated by caspases and calpains also plays a critical role in apoptosis [38, 39].Our results showed a concentration-dependent cleavage of BAX by CuB treatment in MDA-MB-231 and SKBR3 cells. However, the cleavage of BAX was not observed in MCF-7 cells. This is not surprising as MCF-7 is a caspase 3 null cell line [40]. Cleavage of BAX by CuB was significantly blocked by caspase 3 inhibitor. These observations suggest a direct role of caspase 3 in BAX cleavage induced by CuB treatment. However, further studies are required to confirm the mechanism and implications of BAX cleavage by CuB.

Our results indicated TGFβ mediated interaction of HER2 with integrins. HER2 utilizes multiple mechanisms to enhance tumorigenicity. Kalra et. al. have shown that ILK1 regulates HER2 expression by coordinating the molecular functions of TWIST and YB-1 [15]. In agreement, our results showed that CuB treatment inhibited ILK1/YB-1/TWIST signaling, which can explain the down-regulation of HER2 in our model.

Furthermore, the tumorigenicity by EGFR family of receptors is known to be mediated by Src kinase and Src inhibition can lead to suppression of tumorigenicity [14, 47]. Our results demonstrated significant inhibition of Src expression and phosphorylation by CuB treatment. These changes were accompanied with suppression of phosphorylated P130CAS, the downstream effector molecule of Src kinase [48]. Interestingly, TACE dependent ligand shedding is also known to play role in regulating HER2-integrin signaling in breast cancer [16]. We observed suppression of TACE expression by CuB treatment in all the breast cancer cell lines tested. These observations suggest significant influence of CuB treatment on HER2 and related signaling in breast cancer irrespective of genotypes and phenotypes of various breast cancer cells used in our studies.

Integrins are the master regulators of various cellular processes [49]. ITGA6B4 has been recently identified to play a significant role in breast cancer progression through diverse mechanisms [20, 21, 26, 50-53]. ITGB4 is of special prognostic significance in basal type breast cancer exemplified by MDA-MB-231 cells used in this study [54]. Evidence suggests that HER2 is present at the crossroad of integrin signaling in breast cancer [27, 29, 55]. Guo et. al. demonstrated that ITGB4 augments HER2 signaling to promote breast cancer progression, while another study by Wang et. al. demonstrated augmentation of ITGA6B4 by HER2 [28, 30]. Furthermore, a study supports the physical interaction of HER2 with ITGA6, ITGB1 and ITGB4, which can be augmented by TGFβ [16]. These studies also suggest that inhibition of the interaction of HER2 with integrins can be exploited for therapeutic advantage [16]. Our studies confirm the interaction of HER2 with ITGA6. Interestingly, we made a unique observation that TGFβ treatment also increased the constitutive expression of ITGA6, which might be the reason of enhanced interaction of ITGA6 with HER2 by TGFβ treatment. Nonetheless, this interaction was inhibited by CuB treatment. Our observations confirm the interplay between HER2 and integrin α6β4 in breast cancer cells, while suggesting a direct role of TGFβ in enhancing the interaction. Our results also suggest that inhibition of HER2 interaction with these integrins can serve as a powerful mechanism for anti-cancer therapeutics.

In addition to cross-talk with HER2, integrin heterodimers also enhance cell survival by direct signaling [56]. The cytoplasmic domains of heterodimers of β integrins can interact with adaptor proteins like ILK (Integrin linked Kinase) and paxillin to enhance survival signals through AKT and ERK, leading to inactivation of GSK3β and activation of Rho kinases like ROCK1 and RhoA [57, 58]. Our data clearly shows inhibition of ILK1, paxillin, Akt, ERK and Rho kinases while inducing GSK3β by CuB treatment in breast cancer cells. Our time-dependent study indicated that HER2 inhibition was more prominent at 48h of CuB treatment whereas, inhibition of integrins was more at 72h. Therefore, these results suggest that inhibition of integrin followed HER2 inhibition, indicating a link between these pathways.

Interestingly, ITGB1 and ITGB3 have been shown to induce apoptosis through recruitment of caspase8, the process known as integrin-mediated death (IMD) [18]. We observed an increase in the expression of ITGB1, ITGB3 and cleavage of caspase 8 by CuB treatment in MDA-MB-231, SKBR3 and 4T-1 cells, suggesting CuB mediated IMD. However, further studies are required to confirm this mechanism.

Zhang et. al. used 1mg/kg CuB through intraperitoneal injection thrice a week against melanoma tumors. They observed about 50% suppression of tumor growth after 23 days [59]. Kausar et. al. observed that treatment with 1mg/kg CuB every alternate day for 5 weeks reduced the volume of non-small lung carcinoma tumor xenografts by 70% [60]. Furthermore, as shown by Aribi et. al., CuB suppressed MDA-MB-231 orthotopic tumor volume by 42% and 55% at dose of 0.5 and 1mg/kg (i.p.) respectively after 36 days of treatment [61]. In agreement to this study, we also observed a 55% reduction in tumor volumes when 2mg/kg CuB was administered orally every day for 60 days. In addition, in another orthotopic tumor experiment, we employed murine breast cancer cell line 4T-1, which represents late stage aggressive breast carcinoma, and treated the mice with 1mg/kg CuB i.p., every third day [62]. After 28 days, the average tumor volume in treated mice was found to be about 39% reduced as compared to tumors in control mice. Our two different *in vivo* tumor experiments confirm the anti-breast tumor properties of CuB. The tumors of CuB treated mice exhibited inhibitions of HER2/integrins signaling, validating our *in vitro* observations. The two tumor models used in this study displayed significantly different tumor growth kinetics. However, CuB suppressed the tumor growth in both the models by inhibiting HER2/integrin signaling.

Taken together, our results indicate inhibition of HER2-integrin signaling as a novel anti-cancer mechanism of CuB in breast cancer cells. The HER2 inhibition correlates with suppression of ITGA6 and ITGB4, which further lead to inhibition of integrin-mediated cell survival through ILK1 and paxillin. To the best of our knowledge, this is the first report on induction of apoptosis in breast cancer cells through HER2-integrin signaling inhibition.

## MATERIALS AND METHODS

### Ethics Statement

Experiments were conducted in accordance with the ethical standards and according to approved protocol by Institutional Animal Care and Use Committee (IACUC).

### Cell culture

Human breast carcinoma cell lines MDA-MB-231 and MCF-7 were purchased from ATCC and recently authenticated by STR analysis by our core facility. SKBR3 cells were kindly provided by Dr. Terumi Kohwi-Shigematsu (Lawrence Berkeley National Laboratory, Berkeley, CA, USA). These cells were maintained in DMEM supplemented with 10% FBS and 5% PSN [10]. The 4T-1 cells were purchased from Perkin Elmers (Santa Clara, CA, USA). These cells were maintained in RPMI 1640 supplemented with 10% FBS and 5% PSN. All the cells used in this study were within twenty passages after receipt or resuscitation. The cells were maintained and passaged in culture as described by us previously [63].

MCF-7 is an ER positive cell line with wild type p53 while MDA-MB-231 and SKBR3 are ER negative cells and harbor mutated p53. 4T-1 cells are triple negative and p53 null murine cells representing stage IV metastatic breast cancer.

### Cytotoxicity Studies

Cells were plated at a density of 2000-3000 cells/well in 96 well plates and allowed to attach overnight and treated with different concentrations (0-100nM) of Cucurbitacin B (CuB) (Sigma-Aldrich, St. Louis, MO) for various time intervals. The cells were fixed with ice cold 10% trichloroacetic acid, washed and stained with sulforhodamine B (SRB) dye and the optical density was measured in Tris base solution using plate reader, after washing the dye with 1% acetic acid solution as described by us previously [63, 64].

### Annexin-FITC apoptosis assay

Apoptosis assay was performed using a kit (BD Biosciences, CA) according to manufacturer's instructions. Approximately, 0.1x10^6^ cells were plated in 6-well plate and left overnight for attachment. After treatment for 48 and 72h with 0-75nM CuB, cells were harvested after trypsinization, washed and suspended in binding buffer to have cell density of 1x10^6^/ml. A 5.0μl of Annexin V-FITC and 5μl of propidium iodide (BD Biosciences, San Jose, CA) were added to the 100μl of suspension and incubated for additional 20 min at room temperature in dark. Total sample volume was made up to 200μl with binding buffer. Samples were analyzed by flow cytometer after vortexing (Accuri C6, MI).

### Western Blot Analysis

Various breast cancer cells were treated with varying concentrations of CuB (0, 25, 50 and 75nM except for 4T-1 cells, which were treated with 0, 40, 80 and 150nM) for 48 or 72h. The MDA-MB-231 cells were treated with 50nM Q-VD-OPH, a caspase 3 inhibitor (Apoptrol, Enon, OH), 1h prior to treatment with CuB and the cells were collected after 48h. In another experiment, MDA-MB-231 cells were treated with 10ng/ml TGFβ (Peprotech, Rocky Hill, NJ) for 1h prior to the treatment with 50nM CuB for 48h. Whole cell lysates were prepared using 4% (w/v) CHAPS buffer while tumor lysates were prepared by homogenizing the tumors in RIPA lysis buffer. Proteins from control and treated samples were subjected to SDS-PAGE and the segregated proteins were transferred to PVDF membrane. The membrane was developed as described by us previously [63, 65, 66]. All antibodies used in the study were purchased from Cell signaling except HER2 (Abcam, Cambridge, MA), ITGB4 (Santacruz Biotechnology, Dallas, TX), TWIST (Santacruz Biotechnology, Dallas, TX), phospho Akt (S473) (Santacruz Biotechnology, Dallas, TX) and Caspase 3 (Santacruz Biotechnology, Dallas, TX).

### Immune-precipitation

Immune-precipitation was performed as described by us previously [67]. Briefly, 0.5x10^6^ MDA-MB-231 cells were plated in 100 mm petridish and treated with 10ng/ml TGFβ for 1h prior to the treatment with 50nM CuB. After 48h CuB treatment, whole cell lysates were prepared using RIPA buffer and immune-precipitated with HER2 antibody (Abcam, Cambridge, MA). Immune complexes were resolved on SDS-PAGE and immune-blotted for ITGA6.

### Tumor therapy model

Female athymic nude mice (4-6 weeks old) were obtained from Jackson Laboratories (Ban harbor, Maine, USA) and maintained under specific pathogen-free conditions. The use of athymic nude mice and their treatment was approved by the Institutional Animal Care and Use Committee (IACUC), Texas Tech University Health Sciences Center, and the experiments were conducted in strict compliance with the regulations. Mice were given antioxidant-free AIN-76A special diet (TestDiet, Richmond, IN) one week before starting the experiment. Exponentially growing MDA-MB-231 cells were harvested, washed and resuspended in PBS at a density of 50x106 cells per ml with 80% matrigel (BD Biosciences, San Jose, CA). A suspension of 0.1mL containing 5x10^6^ cells was injected in the mammary fat pads of the recipient mice. Tumor volumes and animal weights were measured twice a week as described by us previously [68, 69]. When the tumors reached a size of about 50-80 mm^3^, mice were randomly segregated into two groups with ten mice in each group. Test group of mice received 2mg/kg CuB in PBS by oral gavage every day till day 60, whereas control mice received vehicle alone. Mice were sacrificed on day 60 by CO2 overdose and death was confirmed by cervical dislocation in accordance with IACUC guidelines. The tumors were dissected out aseptically from each mouse, weighed and snap frozen in liquid-nitrogen for western blot analysis.

In another experiment, female BALB/c mice (4-6 weeks old) were obtained from Jackson Laboratories (Ban harbor, Maine, USA) and maintained under controlled humidity and temperature conditions. The use of BALB/c mice and their treatment was approved by the Institutional Animal Care and Use Committee (IACUC), Texas Tech University Health Sciences Center, and the experiments were conducted in strict compliance with the regulations. Exponentially growing 4T-1 cells were harvested, washed and re-suspended in PBS at a density of 1x10^6^ cells per ml with 50% matrigel. A suspension of 0.1mL containing 0.1x10^6^ cells was injected in the mammary fat pads of the recipient mice. Tumor volumes and animal weights were measured every alternate day as described by us previously [68, 69]. When the tumors reached a size of about 30-50 mm3, mice were randomly segregated into two groups with eight mice in each group. The treatment group of mice received 1mg/kg CuB in PBS by i.p. injection every third day till day 28. The control mice received vehicle alone. Mice were sacrificed on day 28 by CO2 overdose and death was confirmed by cervical dislocation in accordance with IACUC guidelines. The tumors were removed aseptically from each mouse, weighed and snap frozen in liquid-nitrogen for further analysis.

### Immunohistochemistry

The immunohistochemistry (IHC) was performed as previously described by our lab [69]. Paraffin-embedded tumor tissues were sectioned into 5μm thick sections using microtome (Leica Microsystems Inc., Buffalo Grove, IL). The sections were deparaffinized and rehydrated by performing three washes of xylene for 5 minutes each, two washes of 100% ethanol for 10 minutes followed by two washes of 95% ethanol for 10 minutes and the sections were given two 5 minute washes in double-distilled water (dH2O). Antigens were unmasked by boiling the sections in 10 mM sodium citrate buffer (pH 6.0) followed by incubating the sections at room temperature for about 30 minutes for cooling down. The slides were washed in dH2O three times for 5 minutes each, then incubated in 3% hydrogen peroxide solution in methanol for 10 minutes followed by two washes in dH2O for 5 minutes each. Tumor sections were then washed twice in wash buffer (PBS with 0.1% Tween-20) for 5 minutes each, blocked in 200 μL of blocking solution (5% goat serum diluted in 1% bovine serum albumin solution in PBS) for 30 minutes at room temperature, and incubated with anti–HER2 (1:150) (Abcam, Cambridge, MA) or anti-ITGB4 antibody (1:150) (Santacruz Biotechnology, Dallas, TX) overnight at 4°C. Next day primary antibody was removed and the sections were washed three times in wash buffer for 10 minutes each followed by incubation with Ultravision ONE HRP polymer (Thermofisher scientific, Fremont, CA) for 30 minutes. Subsequently, sections were washed with wash buffer and incubated with DAB Plus chromogen as per the manufacturer's instructions for 15-20 minutes. The sections were counterstained with hematoxylin and dehydrated by incubating in 95% ethanol followed by two incubations in 100% ethanol for 5 minutes each and final incubation was done in xylene for 5 minutes. The slides were mounted using Permount (Fisher scientific, Fair Lawn, NJ) and analyzed under a phase-contrast Olympus microscope (Olympus America Inc).

### Statistical Analysis

Statistical analysis was performed using Prism 6.0 (GraphPad software Inc., San Diego, CA, USA). Results were represented as means ± SD or S.E.M with minimum value of n=3. Data was analyzed by Student's t-test. Differences were considered statistically significant at p<0.05.

## SUPPLEMENTARY FIGURE


