# Supplementary material for: Inhibition of HER2-integrin signaling by Cucurbitacin B leads to in vitro and in vivo breast tumor growth suppression
Source: Oncotarget. 2014 Feb 25;5(7):1812–28. doi: 10.18632/oncotarget.1743 (PMC4039119; doi:10.18632/oncotarget.1743)
Supplement: Supplementary file 1 [file oncotarget-05-1812-s001.pdf]

Inhibition of HER2-Integrin signaling by Cucurbitacin B leads to *in vitro* and *in vivo* breast tumor growth suppression – Gupta et al

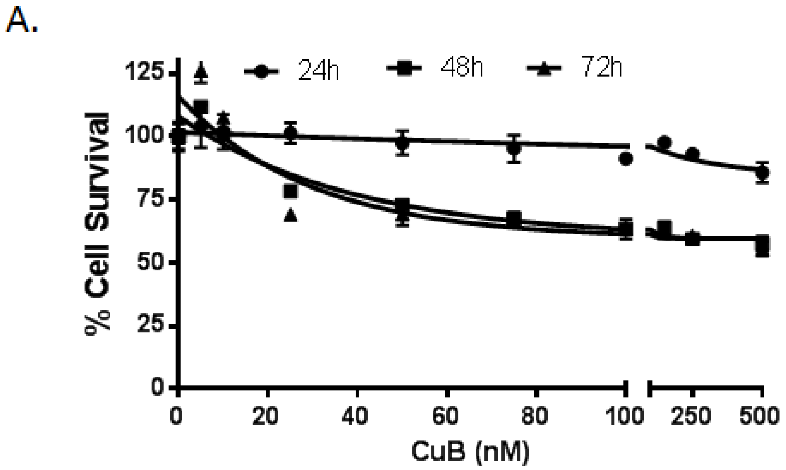

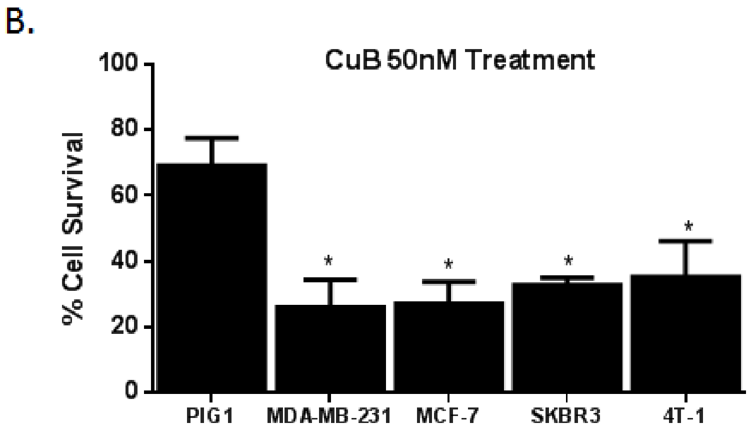

Supplementary Fig 1: A. PIG1 cells were treated with increasing concentrations of CuB for 24, 48h and 72h. Cell survival was measured with sulforhodamine B assay to estimate the IC_{50} values. The experiments were repeated at least three times with 4 replicates in each experiment. B. Comparison of the cell survival of PIG1 and the breast cancer cell lines at 50nM CuB after 72h treatment. *Statistically significant when compared with PIG1 (P<0.05) .
